# Unsupervised Detection of High-Frequency Oscillations Using Time-Frequency Maps and Computer Vision

**DOI:** 10.3389/fnins.2020.00183

**Published:** 2020-03-23

**Authors:** Cristian Donos, Ioana Mîndruţă, Andrei Barborica

**Affiliations:** ^1^Physics Department, Bucharest University, Bucharest, Romania; ^2^Department of Neurology, Bucharest University Emergency Hospital, Bucharest, Romania; ^3^Department of Neurology, Carol Davila University of Medicine and Pharmacy, Bucharest, Romania

**Keywords:** electroencephalogram (EEG), high-frequency oscillations, time-frequency maps, computer vision, signal detection

## Abstract

High-frequency oscillations >80 Hz (HFOs) have unique features distinguishing them from spikes and artifactual components that can be well-evidenced in the time-frequency representations. We introduce an unsupervised HFO detector that uses computer-vision algorithms to detect HFO landmarks on two-dimensional (2D) time-frequency maps. To validate the detector, we introduce an analytical model of the HFO based on a sinewave having a Gaussian envelope, for which analytical equations in time-frequency space can be derived, allowing us to establish a direct correspondence between common HFO detection criteria in the time domain with the ones in the frequency domain, used by the computer-vision detection algorithm. The detector identifies potential HFO events on the time-frequency representation, which are classified as true HFOs if criteria regarding the HFO's frequency, amplitude, and duration are met. The detector is validated on simulated HFOs according to the analytical model, in the presence of noise, with different signal-to-noise ratios (SNRs) ranging from −9 to 0 dB. The detector's sensitivity was 0.64 at an SNR of −9 dB, 0.98 at −6 dB, and >0.99 at −3 dB and 0 dB, while its positive prediction value was >0.95, regardless of the SNR. Using the same simulation dataset, our detector is benchmarked against four previously published HFO detectors. The F-measure, a combined metric that takes into account both sensitivity and positive prediction value, was used to compare detection algorithms. Our detector surpassed the other detectors at −6, −3, and 0 dB and had the second best F-score at −9 dB SNR after the MNI detector (0.77 vs. 0.83). The ability to detect HFOs in clinical recordings has been tested on a set of 36 intracranial electroencephalogram (EEG) channels in six patients, with 89% of the detections being validated by two independent reviewers. The results demonstrate that the unsupervised detection of HFOs based on their 2D features in time-frequency maps is feasible and has a performance comparable or better than the most used HFO detectors.

## Introduction

A pioneering study by Fisher et al. (1992) on five epileptic patients implanted with subdural electrodes showed a significant increase of spectral power above 35 Hz at the beginning of epileptic seizures exhibiting electrodecremental patterns and hypothesized that high-frequency (HF) recordings may be useful in localizing the seizure onset (Fisher et al., 1992).

In the years that followed, many studies revealed that HF oscillations (HFOs) in the 80–250-Hz frequency range, also known as “ripples,” can be identified in the hippocampal and parahippocampal regions of rodents (Buzsáki et al., 1992; Ylinen et al., 1995), primates (Skaggs et al., 2007), and humans (Bragin et al., 1999; Matsumoto et al., 2013). These studies identified HFOs in healthy subjects or in epileptic patients performing visual or motor tasks (Matsumoto et al., 2013), while other studies found an increased number of HFOs in brain regions that are part of the epileptogenic network; therefore, the HFO was considered to be a potential epilepsy biomarker (Urrestarazu et al., 2007; Jacobs et al., 2009; Brázdil et al., 2010; Kerber et al., 2013; Geertsema et al., 2015). However, in spite of the various existing models of HFO generation (Fink et al., 2015; Helling et al., 2015), it is still a matter of debate how to distinguish physiological and pathological HFOs (Engel et al., 2009; Waldman et al., 2018).

Currently, the gold standard of marking HFOs is still based on visual analysis of band-pass filtered signals, as described in detail by Jacobs et al. (2009). It implies the filtering of the electroencephalogram (EEG) signal in the 80–250-Hz frequency range (“ripple” band) or 250–500 Hz (“fast ripple” band), followed by a visual search of portions of the filtered EEG signal that have “clearly visible” higher amplitudes than the rest of the signal. At least four oscillations have to be counted in each HFO (Jacobs et al., 2009; Zijlmans et al., 2017). The main drawback of this method derives from the fact that the human eye cannot accurately evaluate a complex mix of frequencies by looking at the filtered signal, as the high-amplitude low-frequency components of the spectrum may obscure low-amplitude HFOs. Moreover, it has been previously shown that interictal spikes and sharp transients, when filtered in the HFO frequency range of 80–250 Hz, may result in HFO-like oscillations, leading to false HFO detections, and it was suggested that HFOs should be identified using time-frequency analysis (Bénar et al., 2010; Burnos et al., 2014). The distinctive signature of true HFOs is considered to consist in “blobs” of more or less regular shapes that are detached from the time axis and whose centroid is located above 80 Hz (Bénar et al., 2010).

The aims of this report are to: (1) come up with a definition of HFOs in the time-frequency domain, referring to the presence of blobs having centroids located above 80 Hz, by introducing an analytical model that translates the one-dimensional (1D) time-domain definition into the 2D time-frequency domain; and (2) design a detector to identify HFO's signature in 2D time-frequency domain using computer-vision algorithms.

Most automatic HFO detector tool kits use the time-frequency analysis solely for the visual validation of HFO candidates which have been identified using a time-domain detection method (Crépon et al., 2010; Burnos et al., 2014; Amiri et al., 2016; Fedele et al., 2016; Liu et al., 2016). Therefore, HFOs that are not part of the candidates will not be identified in the time-frequency analysis step, resulting in “misses” or “false negatives.” Visual analysis of time-frequency maps provides access to detailed features of the HFOs, allowing for a better discrimination of the true HFOs from the artifactual components (Bénar et al., 2010). We aim at duplicating this complex process of visual detection or validation of the HFOs using time-frequency maps in a computer-based image analysis algorithm, to be used as a high-performance HFO detector. In our study, we provide an unsupervised detection algorithm that combines time-frequency analysis and computer vision to replicate the “visual analysis” of the time-frequency representation. Our algorithm performs the detection directly in the time-frequency space, reducing the number of potential misses. To avoid the limitations and subjectivity of visual markings and the absence of a “ground truth” in real EEG recordings confirmed by time-frequency representations, we validate our automatic detector on simulated EEG signals that contain a mix of discrete HFO events and pink noise (Miyakoshi et al., 2013), with various signal-to-noise ratios (SNRs). We compare the performance of our detector with four previously published detection algorithms (Staba et al., 2002; Gardner et al., 2007; Crépon et al., 2010; Zelmann et al., 2012) using their implementation available in RIPPLELAB (Navarrete et al., 2016). We also validate the detector on intracranial EEG signals recorded during stereo-EEG procedures.

## Methods

### Description of the Workflow for High-Frequency Oscillation Detection

**Figure 1:**
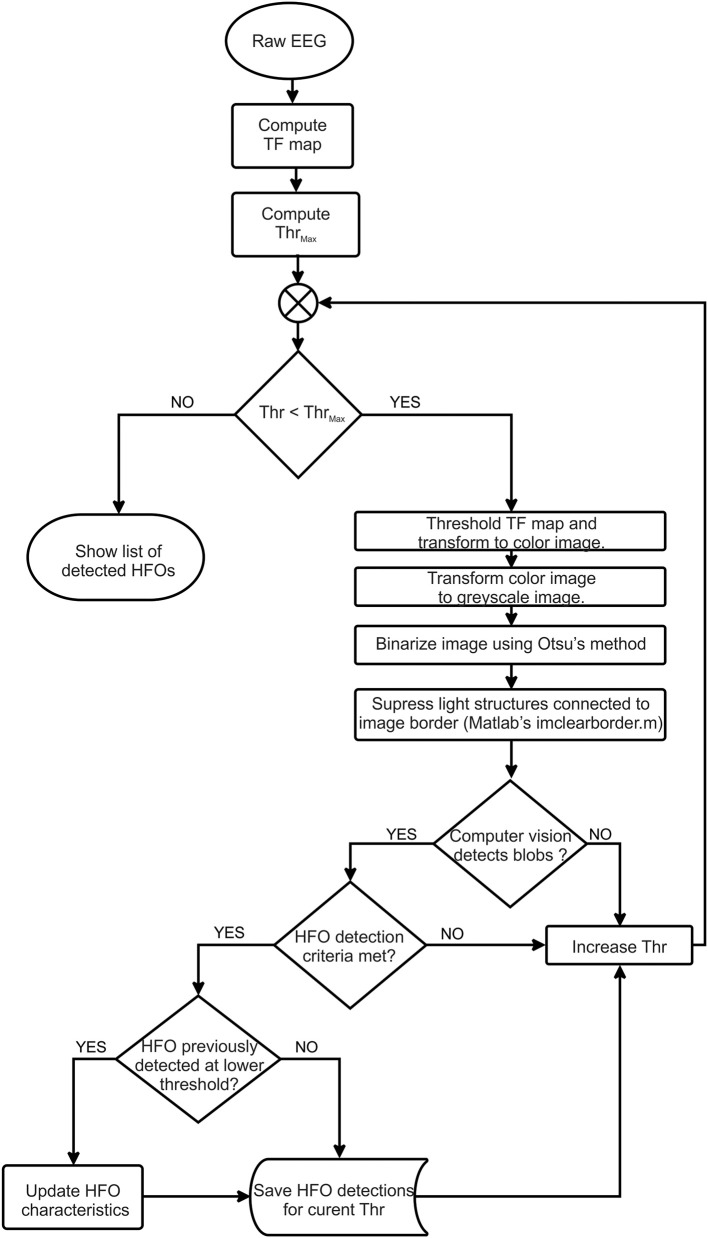
Flowchart of detection algorithm.

The detection algorithm flowchart is shown in Figure 1 and is described in detail in the following sections.

### Analytical Modeling of High-Frequency Oscillations

Simulated signals, based on a general model of HFOs superimposed on spikes or sharp transients (Urrestarazu et al., 2007), are introduced for assessing the detector's performance.

The spikes are considered to have a Gaussian shape, characterized by the spike amplitude (*A*_*spike*_) and the standard deviation (σ_*spike*_). In the time domain, it can be written as:

(1) $$\begin{array}{l} Spike\left(t\right)=A_{spike}e^{-\frac{t^{2}}{2\sigma_{spike}^{2}}} \end{array}$$

The HFO is modeled as a sinewave with a Gaussian envelope:

(2) $$\begin{array}{l} HFO\left(t\right)=\underset{envelope}{\underset{︸}{A_{HFO}\cdot e^{-\frac{t^{2}}{2\sigma_{HFO}^{2}}}}}\cdot \underset{oscillation}{\underset{︸}{\cos\left(2\pi \cdot f_{0}\cdot t\right)}} \end{array}$$

where *A*_*HFO*_ and σ_*HFO*_. are the amplitude and the standard deviation of the Gaussian envelope, and *f*_0_ is the frequency of the sinewave.

Combining (1) and (2), we obtain the formula in the time domain of an HFO superimposed on a spike:

(3) $$\begin{array}{l} x\left(t\right)=Spike\left(t\right)+HFO\left(t\right) \end{array}$$

In the frequency domain, the Fourier transform of the HFO's Gaussian envelope is also a Gaussian:

(4) $$e^{-\frac{t^{2}}{2\sigma^{2}}}\overset{F}{\Leftrightarrow }\sqrt{2\pi }\sigma_{HFO}e^{-2\pi^{2}\sigma_{HFO}^{2}f^{2}}$$

The modulation theorem (Papoulis, 1962):

(5) $$F\{\cos(2\pi f_{0}t)\cdot g(t)\}(f)=\frac{1}{2}\left[G(f-f_{0})+G(f+f_{0})\right]$$

allows us to derive the equation for the frequency spectrum of the HFO:

(6) $$\begin{array}{l} HFO\left(f\right)=A_{HFO}\sqrt{\frac{\pi }{2}}\sigma_{HFO}e^{-2\pi^{2}\sigma_{HFO}^{2}{\left(f-f_{0}\right)}^{2}} \end{array}$$

The Fourier transform of the spike and HFO signal described in the time domain by Equation (3) is therefore:

(7) $$\begin{array}{l} y\left(f\right)=A_{spike}\sqrt{2\pi }\sigma_{spike}e^{-2\pi^{2}\sigma_{spike}^{2}f^{2}}\\ \;+A_{HFO}\sqrt{\frac{\pi }{2}}\sigma_{HFO}e^{-2\pi^{2}\sigma_{HFO}^{2}{\left(f-f_{0}\right)}^{2}} \end{array}$$

**Figure 2:**
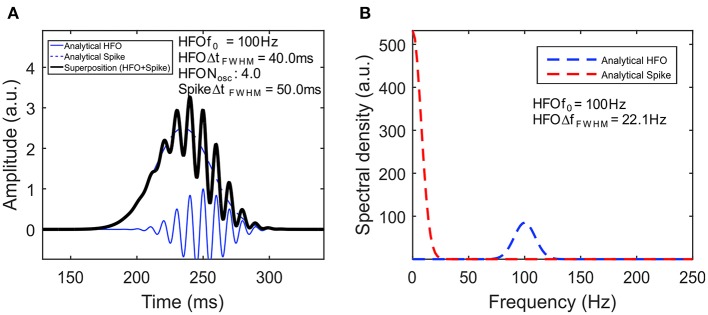
High-frequency oscillation (HFO) model. **(A)** Simulated signal of HFO superimposed on a spike. The continuous blue line represents the HFO, while the dashed blue line represents the spike. Their superposition is shown with a black line. **(B)** The amplitude spectral density of the signal.

Figure 2 visually illustrates a simulated signal based on our model (A) and its frequency spectrum (B). The spike has an amplitude of *A*_*spike*_ = 2.5 au and 50-ms duration at full-width half-maximum (FWHM). The HFO has a frequency of *f*_0_ = 100 Hz, while its envelope has an amplitude of *A*_*HFO*_ = 1 au, 40-ms duration, and four oscillations, both computed at FWHM. To make the model more realistic, we have added a Δ*t* = 15-ms delay to the HFO with respect to the spike, by replacing *t* in Equation (2) with *t* + Δ*t*. One can clearly see in both Equation (7) and its plot in Figure 2B that the spectral components of the spike are represented by a Gaussian centered on the origin, whereas that for the HFO, there is a Gaussian centered at *f*_0_.

### Time-Frequency Maps

The 1D analysis in the frequency domain alone has a number of limitations. First, in the context of EEG signal analysis and visual markings of HFOs, it has been previously shown that notch filtering (Kirac et al., 2016) and high-pass filtering of spikes and sharp transients may produce artifacts that can be easily mistaken as HFOs (Bénar et al., 2010). A second limitation relates to the fact that low-frequency components of the spectrum have a higher amplitude than the HF components, thus restricting the visual analysis to oscillations that are close to the high-pass filtering frequency, which are shadowing higher-frequency lower-amplitude oscillations that may be also present in the EEG signal.

The most commonly used method for computing time-frequency maps is by employing a continuous wavelet transform, which is defined as:

(8) $$\begin{array}{l} x\left(s,\tau \right)=\frac{1}{\sqrt{\left|s\right|}}\overset{\infty }{\underset{-\infty }{\int }}x\left(t\right)\bar{\psi }\left(\frac{t-\tau }{s}\right)dt \end{array}$$

where Ψ is the mother wavelet, s is the scale of the mother wavelet, and τ is the translation of the mother wavelet.

The wavelet transform has been intensively used to study a wide range of neurological diseases (Ahmadlou et al., 2012a,b; Sankari et al., 2012) and can be implemented in applications such as single-unit isolation (Ortiz-Rosario et al., 2015), seizure detection (Li et al., 2016), and brain computer interfaces (Hsu, 2011).

For EEG signal analysis, the Morlet wavelet is perhaps the most commonly used and was found to be particularly useful in HFO analysis (Le Van Quyen and Bragin, 2007). As it has been successfully used in previous studies for both visual validation of manual markings (Bénar et al., 2010; Crépon et al., 2010; Alkawadri et al., 2014; Navarrete et al., 2016) and automatic HFO detectors (Zelmann et al., 2010), we decided to use it in the current study.

**Figure 3:**
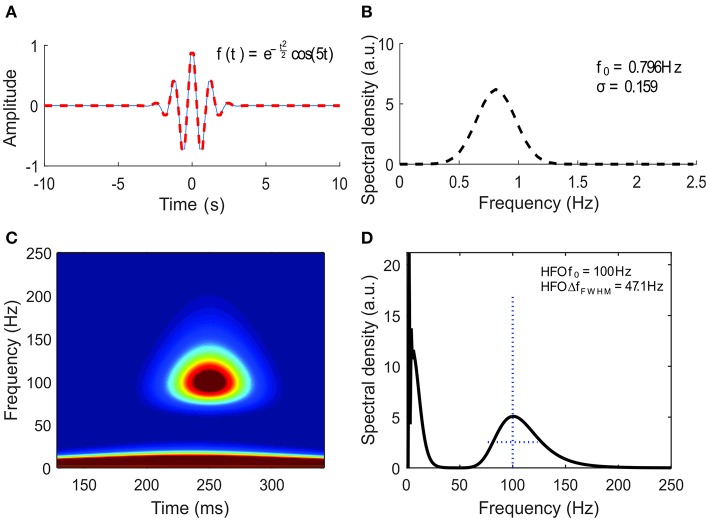
Time-frequency representation of the high-frequency oscillation (HFO) model. **(A)** The Morlet wavelet. **(B)** The frequency spectrum of the Morlet wavelet. (**C**) Time-frequency map of the HFO model defined by Equation (3). **(D)** The instantaneous amplitude spectral density of the HFO.

The Morlet mother wavelet (Figure 3A) has been initially introduced for the analysis of geoseismic signals (Goupillaud et al., 1984) and is a particular case of the more general Gabor wavelet, defined by Equation (9), in which 2π · *f*_0_ = 5(Kumar and Foufoula-Georgiou, 1997). Le Van Quyen et al. (2001) defined the number of cycles as *nco* = ωσ = 2π · *f*_0_.

(9) $$\begin{array}{l} \psi \left(t\right)=\underset{envelope}{\underset{︸}{e^{-\frac{t^{2}}{2\sigma^{2}}}}}\cdot \underset{oscillation}{\underset{︸}{\cos\left(2\pi \cdot f_{0}\cdot t\right)}} \end{array}$$

The frequency spectrum of the Morlet wavelet is shown in Figure 3B. Within the continuous wavelet transform, the Morlet wavelets are scaled so that their envelope's standard deviation fits the duration of various oscillations within the EEG signal.

The time-frequency representation of the analytical signal defined by Equations (1–3) and plotted in Figure 2A containing a prototype HFO superimposed on a spike is shown in Figure 3C. One has to note the appearance of the HFO as a blob that is isolated from the low-frequency components of the spike. A cross section of the time-frequency map, performed at a time corresponding to the peak of the HFO (Figure 3D), shows a spectral density in agreement with the analytical form plotted in Figure 2B. The presence of blobs and their characteristics (amplitude, centroid, FWHM) will be used in the subsequent analysis as the signature of true HFOs.

### Computer Vision for Automatic High-Frequency Oscillation Detection

**Figure 4:**
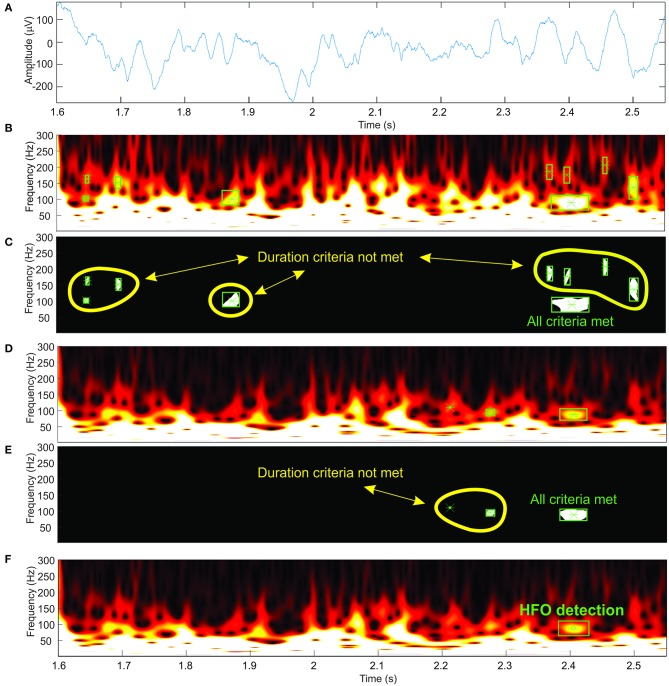
Automatic high-frequency oscillation (HFO) detection. **(A)** Electroencephalogram (EEG) signal. **(B,D)** Time-frequency representations at two different amplitude thresholds. **(C,E)** Detected blobs shown in white. **(F)** HFO detection.

Our automatic HFO detector is based on time-frequency maps that are computed for a specified interval (1 s) of EEG signal (Figure 4A) by performing a continuous wavelet transform. Consecutive windows have a 200-ms overlap that ensures HFO candidates located at the window edges will still be detected in at least one window. A computer-vision algorithm processes each time-frequency map (Figures 4B,D) as an image, attempting to identify blobs that correspond to the presence of the HFOs. This approach aims to automate the visual analysis of time-frequency representations that would be performed by a human. So, in this context, computer vision means all processing steps performed on the time-frequency images that lead to the identification of HFO candidates.

We suppress structures that are lighter than their surroundings and that are connected to the image border (Soille, 2004) (this step is useful to discard any wavelet filtering artifacts located at the extremities of the time window and to eliminate low-frequency components of the signal). Otsu's method allows us to compute a threshold which is further used to convert the image to black and white (Otsu, 1979). We use the black-and-white image to perform blob analysis and detection (Figures 4C,E). The blob analysis is performed using the “vision.BlobAnalysis” function in Matlab's Computer Vision Toolbox. This function analyzes the black-and-white image and, for each white blob resulting after image binarization, computes the blob centroid coordinates, width and height in pixels. Knowing the exact dimensions of the image and the screen's resolution, we can determine the exact location in time, frequency, and the amplitude of each detected blob in the original time-frequency representation (Figure 4F).

### Detection Validation Criteria

Blobs detected in the previous step are considered HFO candidates, which are validated as HFOs using the following criteria:

1. 80 < HFO_frequency_ < 250 Hz (ripples) or 250 < HFO_frequency_ < 500 Hz (fast ripples)
2. HFO_duration_ > N_osc_^*^ 1/HFO_freq_
3. HFO_amplitude_ > 3.5 ^*^ C_thresh_ (adapted from Alkawadri et al., 2014).

where HFO_frequency_ → blob's centroid frequency; HFO_amplitude_ → blob's centroid amplitude (wavelet coefficients); HFO_duration_ → width of the rectangle which contains the blob; N_osc_ = 4.5 → minimum number of oscillations at HFO_frequency_ (adapted from Haegelen et al., 2013, and Jacobs et al., 2010); C_thresh_ → mean of wavelet coefficients in the 80–100-Hz range, computed on time intervals where the wavelet coefficients are less than the amplitude threshold.

### Image Intensity Thresholds

The steps described in previous paragraphs only take into account one intensity threshold of the time-frequency image. As the amplitude of various frequencies within the EEG signal shows large variations, it is required to use a variable image intensity threshold to be able to detect the HFOs in the upper frequency range of the spectrum, which would otherwise be shadowed by the higher amplitudes of the lower-frequency components within the spectrum (Figures 4B,D).

Our algorithm starts with an initial threshold computed as the root mean square (RMS) of the EEG signal for the selected channel, computed during the first 15 s. This step ensures the adaptation of the algorithm to the different EEG amplitudes which are found on different channels.

The threshold is then decreased exponentially starting from an initial threshold using a geometric progression with a common ratio of 0.8:

(10) $$\begin{array}{l} thresh_{i}=0.8^{i-1}\cdot thresh_{0} \end{array}$$

where *thresh*_*i*_ is the *i*-th therm of the geometric progression and *thresh*_0_ is the initial threshold, computed as the RMS of the first 15 s of the EEG signal.

For each image intensity threshold, we repeat the previously described steps. For the decreasing thresholds, the detected HFOs are considered identical and not marked if the rectangle of the newly detected HFO already contains the centroid of a previously detected HFO. In this way, we avoid false large markings which may occur due to the fact that two nearby blobs may become one larger blob at lower thresholds.

The processing steps described above were implemented in Matlab R2016a using the “vision.BlobAnalysis” function in the Computer Vision Toolbox. As the Computer Vision Toolbox requires a license, we have provided a compiled executable version of our detector at https://bitbucket.org/cristidonos/hfodetector. The algorithm performs a detection on a 1-s window signal at 15 different image intensity thresholds in ~1.5–2 s on a laptop (Asus Q324U, Intel® Core™ i7 7500U CPU, 16GB RAM).

### Simulation Data

Three hundred HFO events were simulated using the previously described formula [Equation (2)]. The HFO amplitude was normalized to 1, while the frequency was randomly distributed in the 80–250-Hz range. The HFO duration at FWHM was frequency dependent, so that it will be 5, 6, or 7 oscillations long at the HFO frequency in the simulation dataset. We will refer to the 300 s continuous signal having 2 kHz sampling rate and one HFO every second as our simulated HFO signal.

The choice of pink noise was motivated by the resemblance with the frequency spectrum of real EEG recordings, as well as their previous usage in previously published HFO detection studies (Miyakoshi et al., 2013). Three hundred seconds of pink noise with a sampling rate of 2 kHz were generated. The amplitude of the noise signal was normalized to ± 1 au. We will further refer to this signal as our simulated noise signal.

Four additional signals were obtained by performing a weighted summation of the simulated HFO and the simulated noise signals so that constant SNRs of −9, −6, −3, and 0 dB were obtained. The combined signal (*Sig*_*combined*_) was obtained by multiplying the pink noise (*noise*) by a factor *f* so that the desired SNR (*SNR*_*dB*_) is obtained, according to the formula:

$$\begin{array}{l} Sig_{combined}=Sig_{HFO}+f\cdot noise \end{array}$$

where $f=2^{\left|\frac{SNR_{dB}}{3}\right|}\cdot \frac{RMS\left(Sig_{HFO}\right)}{RMS\left(noise\right)}$ and *SNR*_*dB*_ ∈ {−9; −6; −3; 0}.

The simulation dataset and a Matlab GUI of our detection software are made available for testing at https://bitbucket.org/cristidonos/hfodetector.

### Comparison With Other High-Frequency Oscillation Detectors

Four previously published automatic detectors were used to perform HFO detection on the same simulation dataset using their implementation in RIPPLELAB (Navarrete et al., 2016). As named in RIPPLELAB, the four detectors are Hilbert (HIL) (Crépon et al., 2010), MNI detector (MNI) (Zelmann et al., 2010), Short Time Energy (STE) (Staba et al., 2002), and Short Line Length (SLL) (Gardner et al., 2007). For all four detectors in RIPPLELAB, we set the frequency range to 80–250 Hz and the epoch duration to 300 s. Two additional parameters were tuned for MNI and STE, and we will discuss the reasoning in the Results section. All remaining parameters were used with their default values, recommended by the original papers describing the algorithms and successfully used for similar comparisons on manually annotated EEG data (Zelmann et al., 2010; Navarrete et al., 2016; Roehri et al., 2017). For all detectors, we computed the number of true (TPs) and false positives (FPs), the positive prediction value (PPV), the sensitivity (Sens), and the F-measure. F-measure is defined as $F=2\;\frac{PPV\cdot Sens}{PPV+Sens}$ (or $\frac{TP}{TP+\;\frac{FP+FN}{2}}$, where FN is the number of false negatives).

### Intracranial Electroencephalogram Data

The detection algorithm's performance was also evaluated on intracranial EEG data recorded during slow-wave sleep (Jacobs et al., 2008; Alkawadri et al., 2014) in six patients undergoing stereo-EEG presurgical evaluation for drug-resistant epilepsy in our epilepsy center using the methodology described in Donos et al. (2017). For each patient, we selected a total of six bipolar EEG traces recorded on three contacts located in epileptogenic areas and three contacts located in non-epileptogenic areas, and we performed automatic detection of HFOs on a 5-min EEG segment. The detections were visually inspected independently by two experienced reviewers (CD, AB) based on a combined display of the raw EEG trace, the 80–300-Hz filtered EEG trace, and the time-frequency representation of the signal. Detections that did not meet the standard inclusion criteria (Jacobs et al., 2009, 2012; Zijlmans et al., 2017), including the appearance as blob separated from the time axis on time-frequency maps (Bénar et al., 2010; Roehri et al., 2017) were rejected as FPs. The inter-rater agreement was quantified by Cohen's kappa coefficient (Cohen, 1960).

## Results

### Detector Evaluation

**Figure 5:**
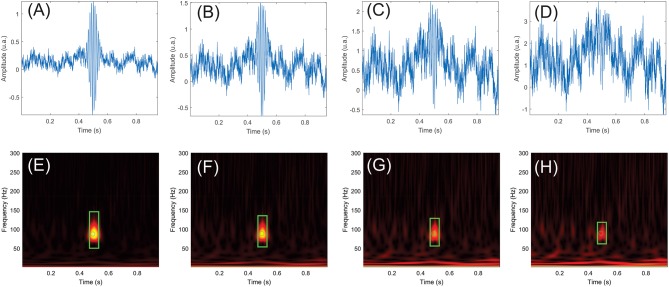
Examples of simulated high-frequency oscillations (HFOs) and pink noise with signal-to-noise ratios (SNRs) of 0, −3, −6, and −9 dB. **(E–H)** show the time-frequency maps of the simulated signals in **(A–D)**. The simulated HFO is centered at 0.5 s in all panels. Events that passed the HFO validation criteria are marked in green. The rectangle represents the duration and frequency extent of the HFO, while the circle shows the HFO's center in time and frequency.

The automatic detection algorithm was run on the simulation dataset. Figure 5 shows a simulated HFO at 88 Hz, superimposed on the noise signal with SNRs of 0 (Figure 5A), −3 (Figure 5B), −6 (Figure 5C), and −9 dB (Figure 5D). The corresponding time-frequency representations are provided in Figures 5E–H.

If multiple candidate events were detected within some limits of tolerance in both time (50 ms) and frequency (5 Hz), they were merged and considered as a single event. Detections within the tolerance limits were compared to the known time and frequency location of each simulated HFO and were considered TPs, while other detections were considered FPs. Whenever multiple detections within the tolerance limits occurred due to detection window overlap and the associated baseline changes, they were considered as unique events.

**Figure 6:**
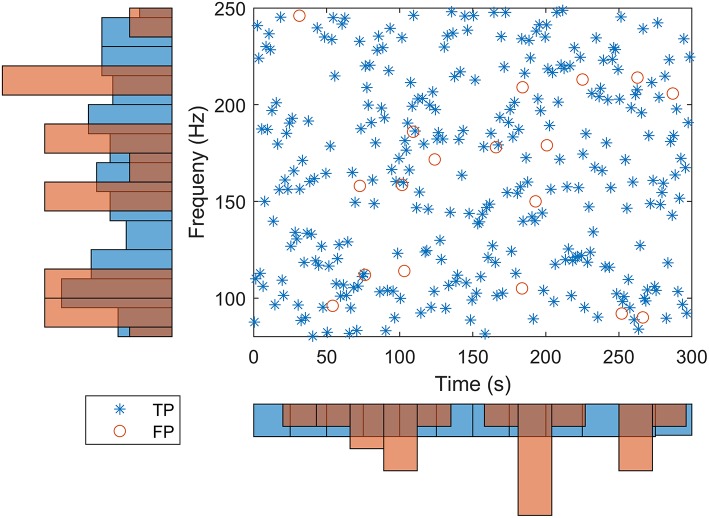
Distribution of high-frequency oscillation (HFO) detections in time and frequency for the simulation dataset. True positive (TP) and false positive (FP) are represented in blue and orange in the scatterplot and in the histograms.

For our detector, the number of TPs ranged between 193 at −9 dB SNR and 299 at −6, −3, and 0 dB SNRs for the mixed simulated HFOs and noise signals. A small number of FPs were detected at all SNRs, with a maximum of 15 FPs at −6 dB SNR. Figure 6 shows the time and frequency distribution of TPs and FPs for all SNRs pooled together. Our detector successfully detected simulated HFOs that cover the whole 80–250-Hz frequency range. The time and frequency localization errors of each simulated HFO were computed as the difference between the ground truth simulated values and the detection values. The average time localization errors and the average frequency localization errors had similar values regardless of the SNR: ~ 5 ms and ~0.8 Hz, respectively (Table 1).

**Table 1:** Detection performance of our detector on the simulation dataset across various SNRs.

**SNR**	**−9 dB**	**−6 dB**	**−3 dB**	**0 dB**	**HFOs only**	**Noise only**
Number of simulated HFOs	300	300	300	300	300	0
TP	193	295	299	299	299	0
FP	7	15	6	2	0	7
PPV	0.965	0.952	0.980	0.993	1.000	0
Sensitivity	0.643	0.983	0.997	0.997	0.997	0
F-measure	0.772	0.967	0.988	0.995	0.998	0
Mean time localization error	0.005	0.005	0.005	0.005	0.005	-
SD time localization error	0.004	0.003	0.003	0.003	0.003	-
Mean frequency localization error	−0.787	−0.832	−0.824	−0.824	−0.824	-
SD frequency localization error	1.281	1.162	1.156	1.156	1.156	-

*FP, false positive; HFO, high-frequency oscillation; PPV, positive prediction value; SNR, signal-to-noise ratio; TP, true positive*.

The PPVs were above 0.95 for all SNRs. Sensitivity and F-measure were 0.64 and 0.77 at −9 dB SNR, but they both increased to more than 0.97 for the remaining SNRs.

The FPs were either simulated HFOs detected outside the time and frequency tolerance limits, either random oscillations in the pink noise that matched the HFO detection criteria by chance.

**Figure 7:**
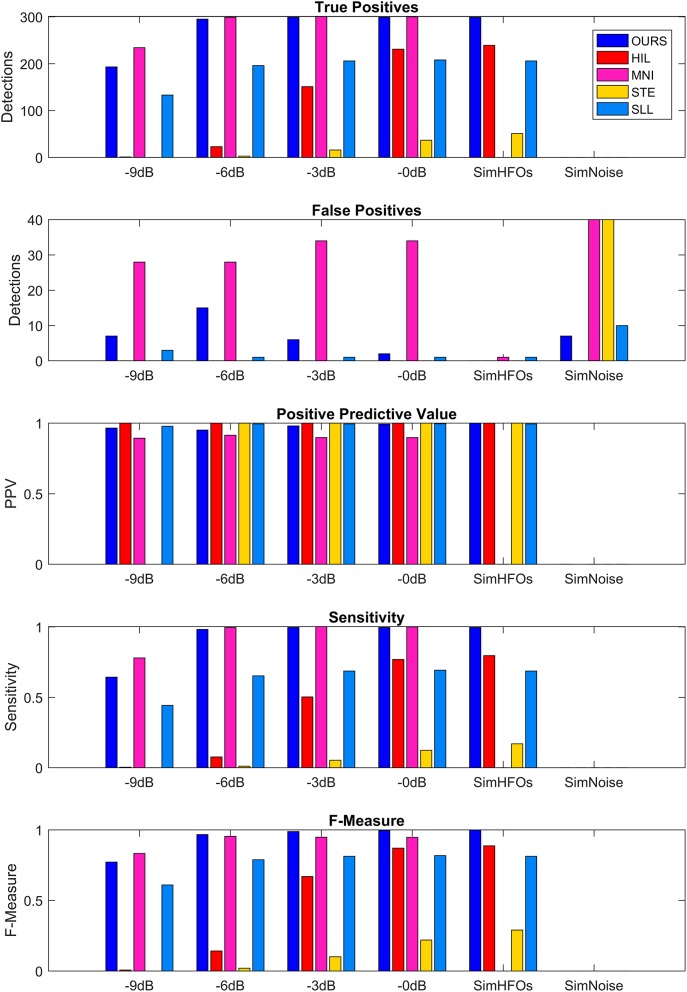
Comparison of detection performance in the simulation dataset. SimHFOs is the signal containing only simulated high-frequency oscillation (HFO), while SimNoise is the signal containing only the pink noise.

The performance metrics of our detector are shown in detail in Table 1 and are compared to four other detection algorithms implemented in RIPPLELAB in Figure 7. Results in Table 1 and Figure 7 are obtained for simulation dataset.

The detector is able to detect HFO events with virtually any frequency by adjusting the frequency criterion (1) described in the Detection Validation Criteria section. In the Supplementary Material, we have validated the detector with an additional dataset of simulated HFOs in the fast ripple band (250–500 Hz), and we obtained similar sensitivity results. The ability to detect even higher-frequency events can be accommodated by adjusting the time and frequency intervals, as well as the resolution of the time-frequency maps, such that the blobs associated with HFOs having the minimal number of oscillations will have a minimal area that will allow the computer-vision algorithm to properly operate.

### Comparison With Other Automatic Detectors

We used the same evaluation scheme as described in Detector Evaluation based on comparing detections to the ground truth of the simulated HFO parameters.

MNI and STE almost failed to detect HFOs with their default parameters. The MNI detector had the initial baseline duration set to 125 s, and using this value, the detection sensitivity and F-measure were <0.05 at all SNRs. After decreasing the baseline duration to 15 s, the detection greatly improved. STE requires explicit definition of the minimum number of oscillations for HFO events. Since all our simulated HFOs had five, six, or seven oscillations, we set the minimum number of oscillations to 4.5, the same value we use for our detector. Additionally, the MNI detector did not detect any HFOs in the absence of noise (SimHFOs). A possible explanation is that the MNI detector relies on a baseline period for detection, which in the absence of noise was maybe too similar to the HFO simulations which were targeted for detection.

Our detector and MNI surpassed all other detectors in terms of sensitivity, F-measure, and number of TP at all SNRs. However, MNI performed slightly better at −9 dB in terms of TPs (234 vs. 193) but also had a significantly larger number of FPs, ranging between 28 vs. 7 at −9 dB and 34 vs. 2 at 0 dB. Consequently, our detector reached higher PPVs at all SNRs and higher F-measures for −6, −3, and 0 dB.

The HIL and SLL detectors had almost similar TP and sensitivities, but overall, we would say that SLL performed better due to the fact that SLL had a sensitivity of 0.44 at −9 db SNR, which increased to 0.69 at 0 dB SNR. HIL had 0 sensitivity at −9 db but quickly increased to 0.07, 0.5, and 0.77 at −6, −3, and 0 dB, respectively. Similar increase trend was observed for F-measure: SLL was more consistent across various SNRs, but HIL reached 0.87 at 0 dB SNR. STE had its highest sensitivity of 0.29 and its highest F-measure of 0.17 at 0 dB. While investigating the causes of such sensitivities, we observed that STE's performance can be improved by reducing the detection threshold from 5 to 2.5 standard deviations, represented by the “RMS SD_threshold” field in RIPPLELAB. However, for the purpose of our simulation, it is important to use the detection parameters already optimized and validated in real EEG data by the authors of each detection algorithm to be able to properly compare performances between various methods.

Our detector had a few FPs at all SNRs, reaching a maximum of 15 at −6 dB. MNI had 28 FPs at −9 and −6 dB SNR. MNI had 34 FPs at −3 and 0 dB, and in the noise only signal. STE had only 34 FPs in the noise only signal. In other cases, no more than 1–2 FPs were detected, regardless of the SNRs. As a consequence, all detectors have a PPV equal or very close to 1.

### Performance on Real Electroencephalogram Data

**Figure 8:**
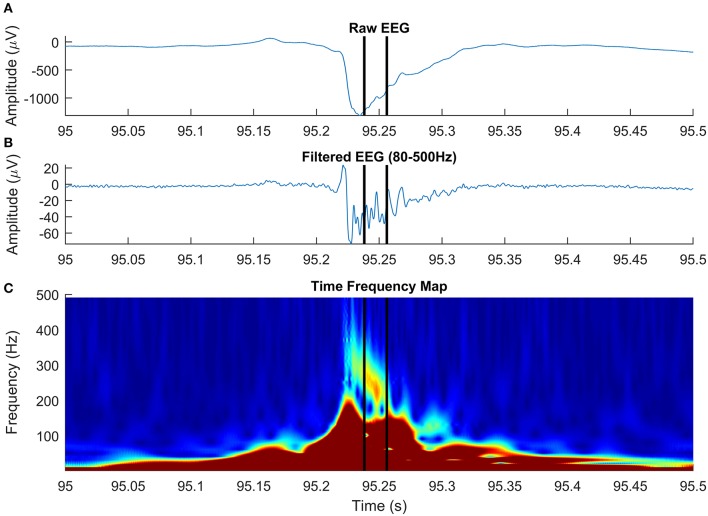
High-frequency oscillation (HFO) with a particular morphology, simultaneous with an epileptic spike, detected on intracranial electroencephalogram (EEG). The panels show raw EEG signal **(A)**, filtered EEG in 80–500-Hz frequency rage **(B)**, and the time-frequency representation **(C)**.

A total of 173 HFOs were detected across all 36 EEG channels recorded intracranially in six patients diagnosed with drug-resistant epilepsy, undergoing presurgical evaluation at the University Emergency Hospital of Bucharest. All patients consented to participate in the study and provide EEG. A detection illustrating the ability of our algorithm to discriminate HFOs having an irregular shape, superiposed on spikes, is shown in Figure 8. After visual validation, 19 events were rejected by at least one reviewer and were classified as FPs, while 154 (89%) events were visually confirmed. Of the 19 events, five were rejected by both reviewers. Cohen's kappa was 0.375, showing a non-accidental fair agreement between the two independent reviewers. The mean number of HFOs per channel was 5.5 ± 6.46, resulting in an average HFO rate of 1.1 events per minute. The mean HFO frequency was 146.72 ± 54.98, and the mean HFO duration was 39 ± 14 ms. It has to be noted that actual EEG recordings have been used in order to create a dataset of real HFOs for validating the detector, not to determine the clinical relevance of the detected HFOs. The study is a purely methodological one that aims to provide an HFO detection method that is built on a mathematical model and is therefore immune to the subjectivity of manual markings. The tool we have developed and made available to the scientific community could be used in the future to better assess the clinical utility of HFOs.

## Discussion

The HFOs are thought to be a correlate of tissue epileptogenicity (Urrestarazu et al., 2007; Jacobs et al., 2009, 2012; Brázdil et al., 2010; Kerber et al., 2013). Being considered a potential biomarker of epileptogenic tissue, HFOs drew a lot of attention from the scientific community. Based on the gold standard of HFO identification, which is the band-pass filtering combined with visual analysis, a large number of automatic HFO detectors emerged (Staba et al., 2002; Gardner et al., 2007; Crépon et al., 2010; Zelmann et al., 2010; Dümpelmann et al., 2012; Gliske et al., 2015). However, all of the detectors mentioned above rely, at least to some extent, on the optimization of the detection against some visually marked HFOs. The subjectivity of visual markings, quantified by the Cohen's kappa coefficient (Cohen, 1960), is reflected by the direct comparison of four automatic detectors (Zelmann et al., 2012), a study in which the median kappa coefficient varied in the 0.05–0.97 range for the four detectors. Indeed, this limitation is also acknowledged by Dümpelmann et al. (2012), which shows that the automatically detected HFOs partially overlap with the visual markings, but nevertheless, the overall HFO rates across channels reflect the patient's epileptogenicity. Considering all of the above, we believe that a standardized model of HFOs is much needed, a model which can be described through mathematical equations and that can be further used to validate automatic detection algorithms. In this study, we provide such an analytical model of the HFO, together with a practical way of identifying the HFOs from real EEG data in an unsupervised manner.

The use of simulated HFOs, whose time localization, duration, and frequency are known, considered to represent the ground truth for evaluating our detection algorithm allows us to overcome the layer of uncertainty caused by the subjectivity of manual HFO markings in real EEG data, for which the ground truth is unknown. Our algorithm showed high sensitivity and F-measure across various SNRs, ranging from 0.64 and 0.77 at −9 dB to 0.997 and 0.995 at 0 dB, respectively. This is a clear proof that our detection algorithm is able to find the HFOs that match the HFO guidelines in terms of frequency and number of oscillations, provided by previous studies (Jacobs et al., 2010; Alkawadri et al., 2014). As a second validation step, we used our HFO detector on intracranial data recorded from patients undergoing stereo-EEG presurgical evaluation for drug-resistant epilepsy. Detections were visually validated by two independent expert reviewers, resulting in 89% of detections being visually confirmed as HFOs. We relied on the fact that our detector had an insignificant number of FPs on simulated data and assumed a similar number of FPs on real EEG data as well. This was indirectly confirmed by the visual validation of the detections and the HFO rates, which were 1.1 events per minute, in agreement other studies (Alkawadri et al., 2014).

The direct comparison on the same simulated dataset between our detector and four other automatic detectors showed that our method provides better sensitivities and F-measures, which are robust in respect to SNR changes. Interestingly, the STE and MNI detectors failed to generally detect the simulated HFOs with their default parameters, which were optimized based on visual markings of real EEG data. While their performance on simulated data was improved by changing some of their parameters, it is very likely that such changes will affect their performance on real EEG. The remaining algorithms, HIL and SLL detectors, had moderate performance across all SNRs.

An interesting observation is that the simulated pink noise contains some “HFO-like” events, which are within the HFO frequency range and meet the other detection criteria defined in the Methods section. The presence of HFO-like events in noise, without electrophysiological substrate, may explain to some extent the modest inter-rater agreements and modest sensitivities that were previously reported in HFO studies (Zelmann et al., 2010; Dümpelmann et al., 2012; Spring et al., 2017).

The four detectors that we compare ours with use indirect ways of detecting and quantifying oscillations in the time domain (line length) or across a frequency range which is biased toward its low edge of the spectrum. These detectors do not heavily rely on the mathematical properties of oscillations, instead, they eventually use empirically derived features tuned to maximize the match between the detections and the visual markings of HFO. A new dataset of simulated HFOs, generated by an innovative method combining an autoregressive model for simulating EEG and discrete wavelet transforms for extracting HFOs from visual markings, was made available by Roehri et al. (2017). However, this dataset also lacks the information about HFO frequencies. Nevertheless, this simulation dataset is used for comparing Delphos, a new detection algorithm with the four other detection algorithms in RIPPLELAB. While such detection algorithms proved to be useful in delineating the epileptogenic areas (Jacobs et al., 2008, 2009; Zijlmans et al., 2011; Burnos et al., 2014; Fedele et al., 2016), it is still a matter debate how to differentiate pathological from physiological HFOs (Engel et al., 2009; Jefferys et al., 2012; Fink et al., 2015). Using our automatic detector, the HFO analysis and the quest for separating pathological and physiological HFOs can extend to another dimension, by using the frequency information.

In agreement with a previous study (Roehri et al., 2017), we conclude that based on our simulated data, optimizing an HFO detector to visual markings is not enough to ensure a robust detection across various SNRs. Paradoxically, detectors that generally failed to detect true oscillations, matching the HFO detection criteria on our simulated dataset, are successful in localizing the epileptogenic focus in real EEG data (Staba et al., 2002; Zelmann et al., 2010). The fact that visually marked (or detected by automatic detectors tuned to visual markings) HFOs are positively correlated to epileptogenic areas may be explained by the high number of other types of interictal events, like spikes and sharp-waves (Engel and da Silva, 2012; Staba, 2012; Zijlmans et al., 2012). These events, known to be associated with the epileptogenic cortex, have an HF content that may be mistaken as HFOs in the filtered signal (Bénar et al., 2010; Amiri et al., 2016). Our unsupervised detector operates directly on time-frequency representations of the EEG signal and looks for “blobs” in the time-frequency space, an HFO landmark, as shown by Bénar et al. (2010). Although the HFO model discussed in this paper is a simplified one, based on HFO “atoms,” our detection algorithm generalizes very well to complex real EEG data, in which HFOs often appear on time-frequency representations as blobs with irregular shape. Figure 8 shows an HFO superimposed on an epileptic spike, centered at 237 Hz, with a duration of 18 ms, that has a particular morphology: it starts at a frequency above 300 Hz and decreases to roughly 220 Hz over ~30 ms. Studying the specific morphology of HFOs may be important in distinguishing pathological from physiological HFOs, although a recent study on a limited subset of four morphological types demonstrated a limited value in delineating the epileptogenic zone (Burnos et al., 2016).

The extent to which HFOs detected on a time-frequency map are correlated with the epileptogenic areas remains to be determined by future clinical studies. In the meantime, we provide additional evidence that there is an inconsistency between the gold standard HFO detection criteria in time-amplitude domain and the actual signal characteristics in time-frequency domain. We come to the conclusion, just like Roehri et al. (2017), that HFO detection criteria need to be revisited by taking into consideration both time and frequency characteristics of HFOs.

## Conclusions

We provide a method for the automatic detection of HFOs that uses a computer-vision approach for detecting the HFO landmarks on time-frequency maps. The algorithm was validated using simulated data, based on an analytical model of the HFOs, as well as intracranial EEG. A mathematical model in time and frequency domain provides a justification of “blobs” unambiguously identifying true HFOs in time-frequency maps. Our detection algorithm was proven to have a high F-measure over a wide range of SNRs.

## Data Availability Statement

The datasets generated for this study are available on request to the corresponding author.

## Ethics Statement

The studies involving human participants were reviewed and approved by Bagdasar Arseni Hospital Ethical Committee approval No 2621/03.02.2012. The patients/participants provided their written informed consent to participate in this study.

## Author Contributions

CD and AB designed the study and performed the analysis. AB wrote the Matlab scripts related to the HFO model and its analysis, while CD wrote the Matlab software related to the HFO detection and its performance analysis. IM provided the clinical data. CD wrote the first draft of the manuscript. All authors contributed to manuscript revision, read, and approved the submitted version.

### Conflict of Interest

The authors declare that the research was conducted in the absence of any commercial or financial relationships that could be construed as a potential conflict of interest.
