# Supplementary material for: Unsupervised Detection of High-Frequency Oscillations Using Time-Frequency Maps and Computer Vision
Source: Front Neurosci. 2020 Mar 23;14:183. doi: 10.3389/fnins.2020.00183 (PMC7104802; doi:10.3389/fnins.2020.00183)
Supplement: Supplementary file 1 [file Data_Sheet_1.pdf]

Supplementary Material

HFOs are being classified as “ripples” if their frequency is in the 80-250Hz range, or “fast ripples” if their frequency is in the 250-500Hz range. The analytical model of the HFO we have introduced in section 2.2 of the paper can describe HFOs, regardless of their frequency. In this supplementary material we present data demonstrating the ability of our computer vision-based detector to detect HFOs in the fast-ripple band.

While in the paper we discuss in detail the HFOs in the ripple frequency band, which are most common, we present here an additional dataset that we use for the validation of fast ripple detection. The new dataset is similar to the one described in section 2.7 of the paper, except it is 30 seconds long and the HFOs are all in the fast ripple frequency range (250Hz – 500 Hz). The simulated data contains one HFO per second.

A sample simulated HFO with frequency in the 250-500Hz range is shown in supplementary figure S1:

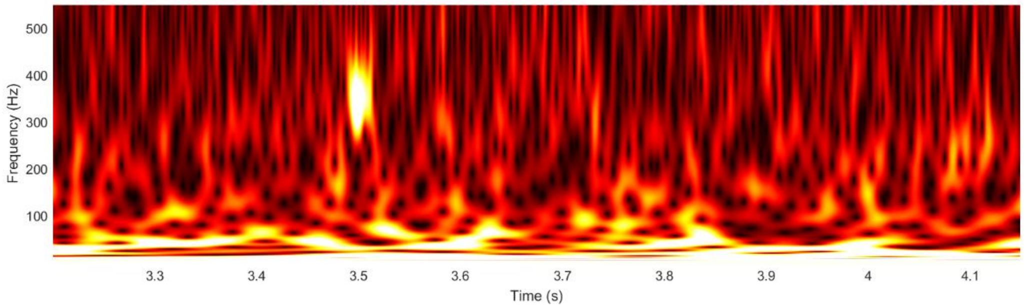

Figure S1. Time-frequency representation of a synthetic HFO having a 337 Hz frequency in the fast-ripple band and -9 SNR level.

To be able to demonstrate detection of HFO events in both ripple and fast ripples bands, we have extended the initial validation criteria from section 2.2 as follows: $\mathbf{80 < HFO_{frequency} < 500\ Hz}$ **.**

We obtained the following detection results for the four signal-to-noise (SNR) ratios we used:

Table S1: Detector correct detections and sensitivity for the dataset containing 30 synthetic HFOs in the fast-ripple band.

SNR (dB)	Correct Detections	% Correct Detections
-9	22	73.3%
-6	29	96.7%
-3	28	93.3%
0	28	93.3%

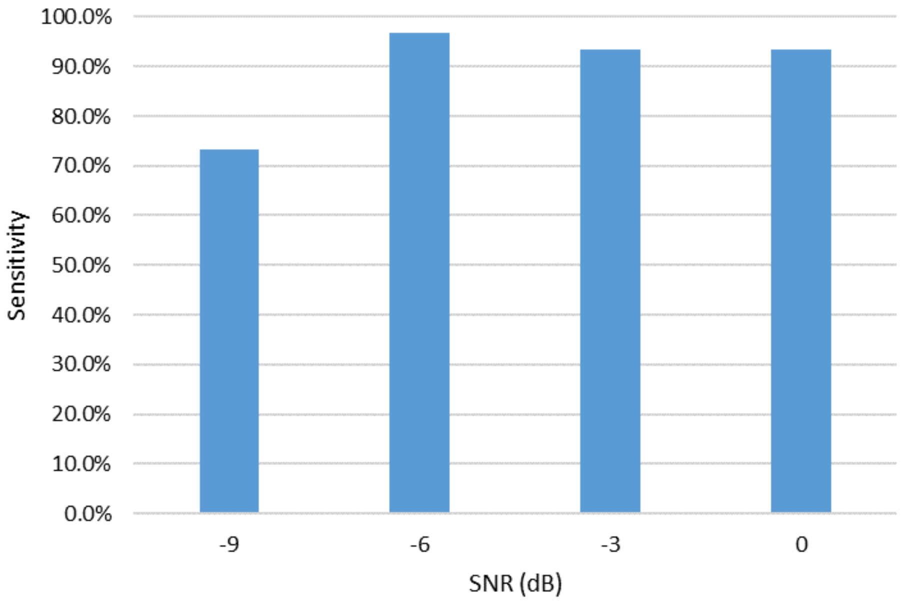

Figure S2. Bar graph representation of the data in Table S1.

By comparing the detector sensitivity data in Table 1 of the paper and in Table S1, one can see that the detector performance for fast ripples detection is similar to the one for ripples.
